# The Impact of Sodium Glucose Co-Transporter 2 (SGLT-2) Inhibitors on Atherogenesis: A Systematic Review of Experimental and Clinical Evidence

**DOI:** 10.3390/life15111784

**Published:** 2025-11-20

**Authors:** Filippo Luca Gurgoglione, Marco Covani, Laura Torlai Triglia, Giorgio Benatti, Davide Donelli, Michele Bianconcini, Emilia Solinas, Iacopo Tadonio, Andrea Denegri, Mattia De Gregorio, Gabriella Dallaglio, Alessandra Dei Cas, Riccardo C. Bonadonna, Luigi Vignali, Giampaolo Niccoli

**Affiliations:** 1 Division of Cardiology, Parma University Hospital, University of Parma, 43126 Parma, Italy; filippolucagurgoglione@gmail.com (F.L.G.); marco.covani@unipr.it (M.C.); laura.torlaitriglia@unipr.it (L.T.T.); gbenatti@ao.pr.it (G.B.); davide.donelli@unipr.it (D.D.); mbianconcini@ao.pr.it (M.B.); esolinas@ao.pr.it (E.S.); itadonio@ao.pr.it (I.T.); adenegri@ao.pr.it (A.D.); mattia.degregorio@unipr.it (M.D.G.); gabriella.dallaglio@unipr.it (G.D.); luvignali@ao.pr.it (L.V.); 2 Department of Endocrinology and Metabolic Diseases, Parma University Hospital, 43126 Parma, Italy; alessandra.deicas@unipr.it; 3 Department of Medicine and Surgery, University of Parma, 43126 Parma, Italy; riccardo.bonadonna@univr.it; 4 Department of Endocrinology, Diabetology and Metabolic Diseases, University of Verona, 37134 Verona, Italy; 5 Department of Medicine and Surgery, University Hospital of Verona, 37134 Verona, Italy

**Keywords:** diabetes mellitus, coronary artery disease, sodium-glucose cotransporter-2 inhibitors, atherogenesis, inflammation, plaque regression, plaque stability

## Abstract

**Background:** Sodium-glucose cotransporter-2 inhibitors (SGLT2i) have demonstrated significant benefits in cardiovascular outcomes trials, but their effect on atherosclerotic plaques remains unclear. This review aims to summarize the current evidence on the impact of SGLT2i on atherogenesis. **Methods:** A systematic search was conducted across PubMed, Embase, Scopus, Web of Science, and Google Scholar databases up to August 2025. Preclinical and clinical studies on the effect of SGLT2i on atherogenesis and atherosclerotic plaque extent and phenotype were included. **Results:** A total of 27 studies were included. Twenty-four studies examined in vitro and animal models of atherosclerosis exposed to SGLT2i, while three studies focused on the effects of SGLT2i on coronary plaques in patients with ischemic heart disease. SGLT2is modulate atherogenesis through multiple mechanisms: prevention and reversal of endothelial dysfunction, reduction in monocyte recruitment and promotion of anti-inflammatory macrophage polarization. Additionally, SGLT2is reduce inflammation and inhibit vascular calcification. Through these mechanisms, SGLT2is decrease plaque burden in both diabetic and non-diabetic atherosclerosis models. Furthermore, they reduce lipid content and macrophages accumulation while increasing fibrous cap thickness, thereby contributing to plaque stabilization. **Conclusions:** Preclinical and clinical evidence suggest that SGLT2is modulate every step of the atherogenic process, reduce atherosclerotic burden and promote coronary plaque stabilization.

## 1. Introduction

Diabetes mellitus (DM) is a metabolic disorder characterized by chronic hyperglycemia, which damages both large and small systemic blood vessels [1]. Accelerated coronary artery disease (CAD) is a hallmark of DM and remains the leading cause of adverse events and mortality in diabetic patients [2,3].

Over the past decade, the management of DM has significantly advanced with the introduction of novel antidiabetic therapies. Sodium-glucose cotransporter-2 inhibitors (SGLT2i), which block glucose reabsorption in the proximal renal tubules, have emerged as a cornerstone of DM management. Despite their modest effect on lowering glycated hemoglobin, these agents have demonstrated significant cardiovascular benefits [4,5]. Randomized controlled trials have shown that the benefits of SGLT2i extend beyond glycaemia control, reducing hospitalizations for heart failure [6,7,8,9] and lowering both cardiovascular and all-cause mortality [10,11,12,13]. The three most extensively studied SGLT2 inhibitors are dapagliflozin, empagliflozin, and canagliflozin. These agents differ in their chemical structure, SGLT2 selectivity, with empagliflozin exhibiting the highest selectivity, and half-life, with dapagliflozin showing the longest duration [4]. Consequently, a uniform class effect among SGLT2 inhibitors cannot be assumed (Supplementary Table S1).

Recent evidence, particularly from preclinical studies, suggests that SGLT2is may also play a beneficial role in attenuating the progression of atherosclerosis and promoting plaque stabilization [4]. Atherosclerosis is a chronic inflammatory disease, and its pathogenesis involves complex molecular mechanisms, including disruption of endothelial cells (ECs) homeostasis, lipid and macrophage accumulation in the subendothelial space, and activation of local and systemic inflammation [14]. These processes contribute to atherosclerotic lesions formation and progression. The composition of atherosclerotic plaques has important prognostic implications: the so-called “vulnerable plaques”, characterized by a large necrotic core, macrophage infiltration, intraplaque hemorrhage, and a thin fibrous cap (FC) [15,16,17,18], are particularly prone to destabilization, leading to acute coronary syndromes (ACS) [14].

This systematic review aims to summarize the current preclinical and clinical evidence on the impact of SGLT2is on cellular components and signaling pathways involved in atherogenesis, as well as their impact on the extent and phenotype of atherosclerotic plaques.

## 2. Materials and Methods

### 2.1. Protocol and Eligibility Criteria

The present systematic review was conducted in line with the PRISMA guidelines [19] and the protocol was registered in the Open Science Framework (https://osf.io/53nrh/overview, accessed on 24 October 2025).

Inclusion and exclusion criteria were based on the following PICOS framework:

P (Population): in vitro and animal models of cellular components and signaling pathways involved in atherogenesis and/or patients with CAD.

I (Intervention): SGLT2i therapy.

C (Comparison): any comparison.

O (Outcomes): alterations in cellular components and signaling pathways involved in atherogenesis and/or changes in atherosclerotic plaque extent and phenotype.

S (Study Design): Preclinical and clinical studies

### 2.2. Search Strategy and Selection Criteria

A comprehensive search was conducted using PubMed, Embase, Scopus, Web of Science, and Google Scholar databases up to August 2025. The full search strategy is detailed in the Supplementary Methods.

**Figure 1:**
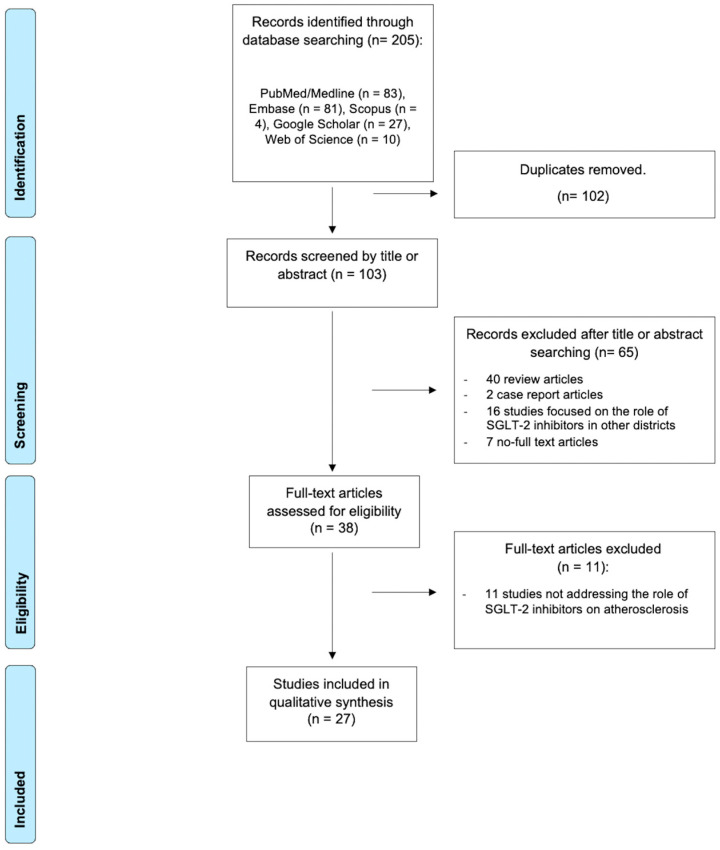
PRISMA flow chart.

Two investigators (F.L.G., M.C.) independently screened titles and abstracts to assess eligibility, performed study selection, and data extraction. In case of discrepancies, data were independently extracted by a third author (G.B.), and then discussed until consensus was reached. The study selection process is illustrated in a flowchart (Figure 1).

### 2.3. Risk of Bias Assessment

The OHAT Risk of Bias Rating Tool was used to assess the quality of in vitro and animal studies, while the NIH Quality Assessment Tool for Observational Cohort and Cross-Sectional Studies was utilized to evaluate the quality of included observational studies. Risk-of-bias assessments were independently conducted by two investigators (F.L.G., M.C.). In case of discrepancies, data were extracted by a third author (G.B.), and then discussed until consensus was reached (Supplementary Tables S2 and S3).

### 2.4. Summary Measures and Synthesis of Results

A qualitative synthesis of the results was conducted to assess the impact of SGLT2i on cellular components and signaling pathways involved in atherosclerosis formation and progression, including ECs homeostasis, monocyte/macrophages pathways, inflammatory pathways, thrombosis, and vascular calcifications. Finally, in vivo studies evaluating the effect of SGLT2i on CAD extent and plaque phenotype were analyzed.

## 3. Results

The search strategy yielded a total of 205 results. Following the article screening process (Figure 1), 27 studies were included in this literature review. Of these, 24 studies employed in vitro and animal models of atherosclerosis exposed to SGLT2i [20,21,22,23,24,25,26,27,28,29,30,31,32,33,34,35,36,37,38,39,40,41,42,43], while 3 studies [44,45,46] investigated the potential impact of SGLT2i therapy in human patients with CAD.

### 3.1. Preclinical Evidence

In vitro and animal studies have demonstrated that SGLT2is can induce both morphological and functional changes in cellular components and signaling pathways involved in atherogenesis, potentially mitigating atherosclerosis formation and progression. Most studies used in vitro cultures or animal models of diabetes or hyperglycemia, whereas only a few explored the effect of SGLT2i on atherogenic processes in a non-diabetic setting (Table 1).

#### 3.1.1. SGLT2i and Endothelial Cells

ECs play a crucial role in maintaining vascular homeostasis. Closely associated with the surrounding glycocalyx and interconnected by protein-binding complexes, these cells form a selective barrier that regulates the translocation of peptides and inflammatory cells. Additionally, ECs modulate vascular tone through the release of vasoactive peptides such as nitric oxide (NO), prostacyclin, endothelin-1, and angiotensin II, and influence blood thrombogenicity and platelet function [47]. Endothelial dysfunction is a key driver of atherosclerosis. The combined effects of hyperglycemia, hypercholesterolemia, and altered shear stress disrupt ECs homeostasis by promoting intracellular oxidative stress [47]. Recent studies suggest that SGLT2is exert protective effects on ECs primarily by reducing intracellular reactive oxygen species (ROS) production.

Semo and colleagues investigated the effect of SGLT2i on ECs under hyperglycemic conditions. Treatment with empagliflozin significantly reduced hyperglycemia-induced ROS production and preserved tissue repair and angiogenesis, by activating the vascular endothelial growth factor and placental growth factor-1 pathways. Notably, the inhibition of intracellular glucose uptake was minimal, suggesting that these effects were largely independent of glucose metabolism [20]. In a subsequent study, administration of SGLT2i (empagliflozin 1 μM, dapagliflozin 1 μM, and canagliflozin 3 μM) significantly decreased ROS production by downregulating the sodium-hydrogen exchanger 1 (NHE1) and NADPH oxidase, while preserving endothelial barrier integrity by preventing vascular endothelial cadherin disruption in a model of human coronary artery ECs subjected to abnormal mechanical stress (10% stretch). These findings were consistent across all tested SGLT2i, supporting the hypothesis of a class-wide antioxidative effect [21,22]. Guo et al. corroborated these observations using human-induced pluripotent stem cell-derived ECs carrying the aldehyde dehydrogenase 2 alcohol flushing variant, which is characterized by enhanced ROS activation, heightened inflammatory pathways, reduced NO bioavailability, and microtubule structure alterations. Specifically, empagliflozin activated the NHE1/AKT kinase/endothelial NO synthase pathway, thereby mitigating oxidative stress and restoring NO bioavailability. Furthermore, SGLT2is have been shown to reduce endothelial barrier permeability by 17–20% through activation of the sirtuin-1/hypoxia-inducible factor-2α pathway [23] and to inhibit high glucose/high fat-induced ECs autophagy [24]. Finally, Ganbaatar et al. performed an elegant study using diabetic apolipoprotein E-deficient (ApoE^−/−^) mice, which demonstrated that 8 weeks of empagliflozin preserved acetylcholine-induced endothelium-dependent vasodilation and reduced inflammation in both ECs and perivascular adipose tissue. Importantly, empagliflozin administration significantly reduced lipid content (*p* < 0.05), macrophage infiltration (*p* < 0.001), and atherosclerotic lesion size in the aortic arch (*p* < 0.01) [25].

#### 3.1.2. SGLT2is and Monocytes/Macrophages Pathways

A well-established correlation exists between the number of circulating monocytes and the risk of CAD [48]. Their migration into the intimal subendothelial layer and subsequent differentiation into macrophages are essential steps in the development of atherosclerotic plaques. Within the subendothelial space, macrophages internalize oxidized low-density lipoproteins, forming the so-called “foam cells”, and orchestrate inflammatory pathways that drive the progression and destabilization of atherosclerotic lesions [14]. The presence of macrophages within the atherosclerotic milieu is a marker of vulnerable and rapidly progressing plaques, which are associated with an increased risk of ACS [16,18]. Recent evidence suggests that imbalances in monocyte and macrophage subpopulations may influence plaque progression and destabilization. Elevated levels of intermediate [49] and/or non-classical monocytes [50] and M1 macrophages [51] have been associated with advanced CAD, increased risk of ACS, and major adverse cardiovascular events (MACE), while M2 macrophages contribute to tissue repair, and promote plaque stability and regression [52].

Emerging evidence suggests that SGLT2is play a protective role in atherogenesis by modulating monocyte and macrophage functions. In a study by Terasaki et al., dapagliflozin reduced macrophage content by 20% and atherosclerotic plaque size by 33% in ApoE^−/−^ mice. Notably, these effects were observed exclusively in the diabetic setting [26]. In a subsequent mechanistic study by the same group, ipragliflozin reduced foam cell formation by 31% in cultured peritoneal macrophages, via suppression of scavenger receptors and concomitant increase in cholesterol efflux from macrophages [27,28]. Additionally, SGLT2is have been shown to induce macrophage autophagy through activation of the AMPK/UNC-like autophagy-activating kinase 1 initiation complex/Beclin1 signaling pathway, while also promoting polarization toward an M2 macrophage phenotype [29]. Building on these findings, Chen et al. conducted a pivotal study in insulin-deficient ApoE^−/−^ mice subjected to a 7-week high-fat diet. Dapagliflozin treatment significantly reduced macrophage burden by 25%, decreased intraplaque lipid content and increased FC thickness by 60%, suggesting a plaque-stabilizing effect [30].

#### 3.1.3. SGLT2i and Inflammatory Pathways

SGLT2is exert pleiotropic anti-inflammatory effects at both systemic and plaque levels, especially in diabetic models of atherosclerosis, through improved glycemic control and enhanced insulin sensitivity. DM involves multiple concurrent inflammatory pathways. Among these, the NLRP3 inflammasome (Nucleotide-binding Oligomerization Domain-Like Receptor Protein 3), a key component of the innate immune system that mediates caspase-1 activation and the secretion of pro-inflammatory cytokines, promotes atherogenesis by driving the release of interleukin (IL)-1β, IL-6, and IL-18 from activated macrophages, ECs, and vascular smooth muscle cells (VSMCs). Hyperglycemia further aggravates this process by enhancing monocyte recruitment to atherosclerotic plaques by upregulating the monocyte chemotactic protein 1 (MCP-1) and by inducing mitochondrial dysfunction in ECs and macrophages [31].

In a study involving ApoE^−/−^ mice on a high-fat diet and treated with streptozotocin, 12 weeks of dapagliflozin treatment lowered the production of ROS, potent activators of the NLRP3 inflammasome, resulting in reduced serum and aortic root levels of NLRP3 (*p* < 0.01), IL-1β (*p* < 0.05), and IL-18 (*p* < 0.05) [30]. Consistent with these findings, Nakatsu et al. reported that one week of luseogliflozin administration suppressed the hyperglycemia-induced release of tumor necrosis factor-α (TNF-α), IL-1, and IL-6 [32].

Empagliflozin has also been shown to improve insulin sensitivity through modulation of the Akt–GSK-3β pathway, and to attenuate the production of high-sensitivity C-reactive protein (CRP), TNF-α, IL-6, and MCP-1, while reducing M1 macrophage infiltration in both visceral and perivascular adipose tissue [33]. In peritoneal macrophage cultures, Iwamoto et al. observed that tofogliflozin modulated hyperglycemia by inhibiting macrophage accumulation and downregulating IL-1β and IL-6 expression, resulting in a significant reduction in atherosclerotic plaque burden. However, tofogliflozin did not prevent atherosclerotic progression in non-diabetic ApoE^−/−^ mice [34].

An elegant study by Lee et al. was the first to demonstrate anti-inflammatory effects of SGLT2i in a non-diabetic context. In normoglycemic rabbits, dapagliflozin significantly reduced intraplaque levels of TNF-α, IL-1β, and IL-6 without altering glycemic and lipid profiles. Furthermore, dapagliflozin treatment led to a significant reduction in the atheroma burden (38.51 ± 3.16% vs. 21.91 ± 1.22%, *p* < 0.01), lipid accumulation (18.90 ± 3.63% vs. 10.20 ± 2.03%, *p* = 0.047), and macrophage infiltration (20.23 ± 1.89% vs. 12.72 ± 1.95%, *p* = 0.01) [35]. More recently, Xu et al. reported that dysfunctional macrophages, ECs and VSMCs exposed to empagliflozin underwent autophagy via the AMPK signaling pathway, leading to reduced foam cell formation and decreased inflammatory cells recruitment within atherosclerotic lesions [36]. Finally, Lin and colleagues demonstrated that empagliflozin treatment in diabetic db/db mice (C57BLKS/J-leprdb/leprdb) reduced myocardial and perivascular fibrosis, decreased coronary artery wall thickness and improved vascular relaxation capacity [37].

#### 3.1.4. SGLT2i and Thrombosis

Intraluminal thrombus formation is a crucial event in ACS. The destabilization of atherosclerotic plaques serves as the primary trigger for thrombus formation by exposing highly thrombogenic proteins, such as collagen and von Willebrand factor, to the bloodstream [14]. This exposure leads to platelet activation, characterized by morphological and functional changes, including increased expression of CD62p and αIIbβ3 receptors on the platelet surface. These changes promote platelet aggregation and activate the coagulation cascade, ultimately resulting in the formation of an intraluminal clot [53]. The potential impact of SGLT2i on thrombus formation remains debated. Spigoni et al. demonstrated that the addition of empagliflozin or dapagliflozin to platelets purified from the peripheral blood of healthy donors and activated with adenosine diphosphate reduced platelet activation. This effect was attributed to the downregulation of NHE1, an enzyme involved in the expression of CD62p and αIIbβ3 receptors on the platelet surface [38]. Additionally, a pilot clinical study observed an inhibitory effect of empagliflozin on platelet reactivity in 20 diabetic patients with chronic coronary syndromes undergoing dual antiplatelet therapy [39].

In contrast, Liberale and colleagues found no association between SGLT2i therapy and platelet function. Particularly, ex vivo platelet aggregometry assessments revealed comparable collagen- and thrombin-induced platelet aggregation between the empagliflozin and placebo groups. Moreover, three months of empagliflozin therapy had no effect on plasma levels of tissue factor or plasminogen activator inhibitor-1, nor on the time to arterial occlusion [40].

#### 3.1.5. SGLT2 and Vascular Calcifications

Vascular calcifications are frequently observed in patients with DM and chronic kidney disease, with prevalence increasing with age [54]. Their pathogenesis involves several cellular and molecular pathways. A primary mechanism is the trans-differentiation of VSMCs into osteoblast-like cells, able to release osteogenic peptides, such as osteocalcin, osteopontin, and alkaline phosphatase [55]. Additionally, M1 macrophages contribute to vascular calcifications by secreting osteogenic peptides and extracellular vesicles containing hydroxyapatite crystals. Finally, apoptotic and necrotic debris released from dying macrophages also play a crucial role [56].

SGLT2is have been shown to attenuate the progression of atherosclerotic calcifications. Chen et al. conducted a landmark study exploring the effects of canagliflozin in murine models subjected to nephrectomy and vitamin D3 overload to induce vascular calcifications. Treatment with canagliflozin significantly reduced aortic calcium content, as assessed by micro-computed tomography and histopathological analysis, compared with controls. The underlying mechanisms were linked to the ability of canagliflozin to inhibit osteogenic differentiation of VSMCs in a dose-dependent manner by blunting the caspase-1/IL-1β and phospho-NF-κB signaling pathways. This inhibition resulted in decreased levels of osteoprotegerin and bone morphogenetic protein 2, along with increased expression of VSMCs markers such as α-smooth muscle actin [41]. A subsequent study by the same group revealed that canagliflozin also suppresses thioredoxin domain-containing 5, a protein involved in endoplasmic reticulum stress, thereby promoting proteasomal degradation of runt-related transcription factor 2, a critical mediator of VSMCs transdifferentiation [42]. More recently, Li et al. showed that the inhibition of the sirtuin-1/hypoxia-inducible factor-2α pathway by SGLT2i contributes to the prevention of vascular calcification [43].

### 3.2. Clinical Evidence

Recent observational clinical studies have investigated the impact of SGLT2i on the extent and phenotype of coronary atherosclerotic plaques by leveraging intracoronary optical coherence tomography (OCT), the most reliable tool for addressing in vivo atherosclerotic plaque composition [57,58,59] (Table 2).

Sardu and colleagues conducted an observational study involving 369 patients with DM and non-obstructive CAD (20–49% luminal stenosis and a negative functional assessment [fractional flow reserve > 0.80]). The patients were stratified into two groups: those receiving SGLT2i therapy (n = 111) and controls (n = 268). At the 1-year follow-up, the SGLT2i group exhibited a lower inflammatory burden, characterized by reduced expression of NLRP3 and decreased serum levels of caspase-1, IL-1β, CRP, IL-6, TNF-α, CD86 (a marker of M1 macrophages), along with higher levels of CD206, a marker of M2 macrophages. The SGLT2i group also showed greater metabolic improvements, as evidenced by lower body mass index and glycated hemoglobin levels compared with controls. OCT assessment revealed that the SGLT2i group had a significantly higher minimum FC thickness (170.29 ± 23.88 μm vs. 163.19 ± 18.98 μm, *p* = 0.003), a reduced lipid arc (96.17 ± 30.49° vs. 109.82 ± 23.17°, *p* = 0.002), and a lower macrophage grade (6.97 ± 2.35 vs. 9.60 ± 2.88, *p* = 0.001), suggesting a more stable plaque phenotype. Finally, the SGLT2i group experienced a lower rate of MACE (10.8% vs. 22.1%, *p* < 0.05). These results were consistent across different SGLT2is (empagliflozin, canagliflozin, and dapagliflozin), suggesting a potential class effect [44].

In another study, Kurozumi et al. enrolled 109 patients with DM and ACS, stratified into an SGLT2i (n = 40) and a control group (n = 69). An OCT subanalysis was performed on 29 patients (15 in the SGLT2i group) to assess potential qualitative changes in non-stented coronary lesions between the index event and follow-up. At 6 months, SGLT2i therapy resulted in a significantly thicker FC (48 ± 15 μm vs. 26 ± 24 μm, *p* = 0.005), a greater reduction in lipid arc (−29 ± 12° vs. −18 ± 14°, *p* = 0.028), and a larger percentage decrease in total lipid arc (−35 ± 13% vs. −19 ± 18%, *p* = 0.01). At 1 year, the SGLT2i group had a significantly lower occurrence of MACE (hazard ratio 4.72 [1.08, 20.63], log-rank *p* = 0.023), primarily due to a reduced rate of coronary revascularization, compared to the control group [45]. Finally, in a longitudinal computed tomography angiography study, patients treated with SGLT2 inhibitors (SGLT2i) demonstrated a reduction in coronary plaque volume, particularly in non-calcified plaques, after a median follow-up of 14.6 months compared with controls. This finding remained consistent regardless of age, sex, multiple cardiovascular risk factors, or concomitant medications [5,46].

In summary, these three studies clearly delineate the anti-atherosclerotic effects of SGLT2i, resulting in plaque regression and stabilization [44,45,46]. Moreover, two studies have reported a reduction in hard clinical endpoints during follow-up among patients treated with SGLT2i [44,45]. However, the causal relationship between these findings and plaque stabilization remains uncertain, owing to the observational nature of the available evidence.

## 4. Discussion

This systematic review, which includes both experimental and clinical studies, examines the impact of SGLT2i on cellular components and signaling pathways involved in atherogenesis, as well as their effects on the extent and phenotype of atherosclerotic plaques.

The main findings of the study suggest that SGLT2is may: (i) modulate several steps of the molecular pathways implicated in the atherogenic process; (ii) reduce the burden of atherosclerotic lesions; (iii) stabilize the phenotype of atherosclerotic plaques.

SGLT2is have been shown to prevent and reverse endothelial dysfunction both in vitro and in animal models, primarily by suppressing hyperglycemia-induced ROS production [20,21,22]. Furthermore, these drugs help maintain the integrity of the endothelial barrier and restore functional pathways involved in endothelial homeostasis, including NO-mediated vasodilation, angiogenesis, and tissue repair processes [23,24,25]. SGLT2is also modulate macrophage activity by reducing monocyte recruitment and proliferation within atherosclerotic plaques and promote macrophage polarization towards the anti-inflammatory M2 phenotype [26,27,28,29,30]. In addition, SGLT2is exert pleiotropic anti-inflammatory effects at both systemic and intraplaque levels. The primary mechanism involves mitigating diabetic inflammation by inhibiting hyperglycemia-induced damage pathways and restoring insulin sensitivity, which leads to lower levels of inflammatory markers in the serum, visceral and perivascular tissues, as well as within the atherosclerotic plaques [31,32,33,34]. Notably, emerging evidence suggests that SGLT2i may exert anti-inflammatory effects also in normoglycemic rabbits, although the specific pathways remain to be fully elucidated [35]. This body of evidence clearly demonstrates that SGLT2is exert protective anti-atherogenic effects through multiple mechanisms, including suppression of ROS generation, inhibition of the NHE1, and downregulation of NLRP3 inflammasome activation. These protective actions are primarily mediated by direct vascular and cellular effects observed in both hyperglycemic and normoglycemic models [21,22,23,24,25], and are partly attributable to improvements in metabolic homeostasis.

Finally, SGLT2i therapy might prevent the progression of atherosclerotic calcification inhibiting the osteogenic differentiation of VSMCs [41,42,43]. However, at the present the implications of coronary calcification remain incompletely understood. While a high burden of coronary calcifications is associated with an increased risk of cardiovascular events, extensive calcification at the plaque level appears to correlate with a more stable phenotype [60,61]. In this context, inhibition of VSMC osteogenic differentiation in the early stages of plaque development may prevent the formation of spotty calcifications, a phenotype more closely related with vulnerability [62].

Collectively, these multifaceted effects may lead to a reduction in the atherosclerotic burden after SGLT2i therapy, as evidenced by a decrease in the number and extent of atherosclerotic lesions in both diabetic and normoglycemic models of atherosclerosis [30,35,37].

Recent preclinical and clinical studies also suggest that SGLT2i may exert a plaque-stabilizing effect, as evidenced by a reduction in lipid content and macrophages accumulation, along with increased FC thickness within the atherosclerotic lesions. These benefits may be attributed to both metabolic improvements and anti-inflammatory effects. The consistency of results across different tested SGLT2i further suggests a potential class-effect of the SGLT2i. Such plaque-stabilizing properties may ultimately help reduce the risk of plaque destabilization and major adverse cardiovascular events [44,45].

However, these findings are derived from observational studies, which are inherently limited by potential sources of bias. Therefore, a definitive demonstration that the reduction in MACE associated with SGLT2i is causally linked to plaque regression and stabilization still requires confirmation through dedicated randomized clinical trials.

Despite these promising results, several questions remain unanswered. While the anti-atherogenic effects of SGLT2i in diabetic conditions are well-established, their impact in non-diabetic context remains less clear and mechanisms underlying the anti-inflammatory properties of SGLT2i in normoglycemic conditions are not fully understood. Few studies have specifically investigated the effects of SGLT2i under normoglycemic conditions. Although current findings are promising, larger and dedicated studies are warranted to determine whether SGLT2is exert a direct anti-atherosclerotic effect independent of glycemic status.

Finally, the effects of SGLT2i on platelet function and the coagulation cascade as well as other vascular districts [63] remain controversial and require further investigations.

### Limitations

The main limitation of the present review arises from the small number of included clinical studies and the lack of randomized controlled trials. In vitro and animal models of atherosclerosis were heterogeneous and mainly involved diabetic models of atherosclerosis. In some studies, the direct involvement of SGLT2 in the observed response was not tested and the effect of SGLT2i on oxidative stress remains to be fully elucidated. Lastly, the expression of SGLT2 in various cells may differ between species, therefore these data might not be generalized.

## 5. Conclusions

Current preclinical and clinical evidence indicates that SGLT2is exert pleiotropic anti-atherosclerotic effects, acting on multiple cellular and molecular pathways that contribute to atherosclerosis initiation and progression. By modulating endothelial function, macrophage polarization, inflammatory signaling, thrombus formation, and vascular calcification, SGLT2is appear to reduce the overall atherosclerotic burden and promote a more stable plaque phenotype. Future randomized controlled trials, including both diabetic and non-diabetic patients with CAD, are warranted to confirm these findings and to determine whether the anti-atherosclerotic and plaque-stabilizing properties of SGLT2i can ultimately translate into improved cardiovascular outcomes.

## Figures and Tables

**Table 1:** Summary characteristics of the included in vitro and animal studies.

First Author, Date, Reference	Study Design	SGLT2i Therapy	Main Results
Endothelial Cells			
Semo, 2023 [20]	Primary human monocytes, HUVECs, HCAECs, HPECs under hyperglycemia	Empagliflozin 100 ng/mL	Restored PlGF-1 signaling in monocytes and VEGF-A pathway in ECs (in vitro study)
Li, 2021 [21]	HCAECs exposed to 10% stretch	Empagliflozin 1 μM, Dapagliflozin 1 μM, Canagliflozin 3 μM	Reduced stretch-induced ROS production and improved barrier integrity (in vitro study)
Pawlos, 2023 [22]	HUVECs exposed to 25-hydroxycholesterol (10 μg/mL)	Empagliflozin 1 μM, Dapagliflozin 1 μM, Canagliflozin 1 μM	Restored endothelial integrity and VE-cadherin expression (in vitro study)
Lin, 2025 [24]	HUVECs exposed to high glucose	Dapagliflozin 1–10 μM	Reduced EC autophagy (in vitro study)
Ganbaatar, 2020 [25]	Male streptozotocin-induced diabetic ApoE^−/−^ mice	Empagliflozin 20 mg/kg/day orally administered	Improved acetylcholine-induced vasodilation; reduced oxidative stress (in vitro study)
Guo, 2023 [23]	hiPSC-derived ECs carrying *ALDH2* variant	Empagliflozin 10 mg/kg/day intraperitoneally administered	Reduced oxidative stress, decreased endothelial barrier permeability (in vitro study)
Monocytes/macrophages pathways			
Terasaki, 2015 [26]	Streptozotocin-induced diabetic ApoE^−/−^ and db/db mice	Dapagliflozin or Ipragliflozin 1.0 mg/kg/day orally administered	Reduced macrophage infiltration in diabetic mice (animal model: aortic atherosclerotic lesions)
Terasaki, 2017 [27]	Streptozotocin-induced diabetic ApoE^−/−^ and db/db mice	Ipragliflozin 1.0 mg/kg/day orally administered	Reduced foam cell formation (animal model: peritoneal macrophages)
Pennig, 2019 [28]	LDLR/SRB1 streptozotocin-induced diabetic mice	Empagliflozin 35 mg/kg/day orally administered	Decreased lipid content and CD68+ macrophages (animal model: aortic atherosclerotic lesions)
Chen, 2023 [29]	Intraplaque macrophages and female ApoE^−/−^ mice	Canagliflozin 10 μM (in vitro) or 10 mg/kg/day orally	Promoted autophagy (animal model: aortic atherosclerotic lesions)
Chen, 2022 [30]	Streptozotocin-induced diabetic ApoE^−/−^ mice	Dapagliflozin 25 mg/kg/day orally administered	Increased collagen and decreased macrophages (animal model: aortic atherosclerotic lesions)
Inflammation pathways			
Leng, 2016 [31]	Streptozotocin-induced diabetic and non-diabetic ApoE^−/−^ mice	Dapagliflozin 1.0 mg/kg/day intragastrically administered	Reduced macrophage infiltration and interleukin production (animal model: aortic atherosclerotic lesions)
Nakatsu, 2017 [32]	Streptozotocin-induced diabetic and non-diabetic ApoE^−/−^ mice	Luseogliflozin (dose not specified)	Reduced TNF-α, IL-1β, IL-6, MMP-2, MMP-9 release in diabetic mice (animal model: aortic atherosclerotic lesions)
Han, 2017 [33]	ApoE^−/−^ mice	Empagliflozin 1 or 3 mg/kg	Reduced TNF-α, IL-6, MCP-1 levels in serum and adipose tissue (animal model: aortic arch and aortic valve area)
Lee, 2020 [34]	Rabbit model of atherosclerosis from abdominal aorta	Dapagliflozin 1 mg/kg/day orally administered	Reduced macrophage infiltration; increased M2 macrophage subtype (animal model: aortic atherosclerotic lesions)
Iwamoto, 2022 [35]	Streptozotocin-induced diabetic and non-diabetic ApoE^−/−^ mice	Diet containing 0.005% tofogliflozin	Reduced IL-1β and IL-6 expression in peritoneal macrophages (animal model: heart and aortic atherosclerotic lesions)
Xu, 2024 [36]	In vitro macrophages, HASMCs, HUVECs	Empagliflozin 50 μM	Induced autophagy via AMPK pathway (animal model: aortic atherosclerotic lesions)
Lin, 2014 [37]	Male db/db mice	Diet containing 0.03% Empagliflozin	Reduced cardiac fibrosis and coronary wall thickening (animal model: heart and aortic atherosclerotic lesions)
Thrombosis			
Spigoni, 2020 [38]	Human myeloid cells and platelets exposed to stearic acid	Empagliflozin or Dapagliflozin 1–100 μM	Reduced platelet activation (in vitro study: peripheral blood of healthy subjects)
Liberale, 2023 [40]	C57BL/6 mice treated with LPS before carotid thrombosis	Empagliflozin 25 mg/kg	No effect on platelet aggregation, PAI-1, or tissue factor expression (in vitro study: C57BL/6 mice)
Vascular Calcification			
Chen, 2023 [41]	Mouse aortic cells with in vitro–induced calcification	Canagliflozin 5–20 μM	Reduced arterial calcification in VSMCs (animal model: aortic atherosclerotic lesions)
Wu, 2024 [42]	C57BL/6J mice	Dapagliflozin 5–20 μM	Reduced calcification in VSMCs and in vivo aorta (animal model: aortic atherosclerotic lesions)
Li, 2024 [43]	C57BL/6J mice	Dapagliflozin 2.5–10 μM	Inhibited vascular calcification (animal model: aortic atherosclerotic lesions)

Abbreviations: *ALDH2*, Aldehyde dehydrogenase 2; AMPK, AMP-activated protein kinase; ApoE^−/−^, Apolipoprotein E-deficient; CD68, Cluster of differentiation 68; ECs, Endothelial cells; HASMCs, Human aortic smooth muscle cells; hiPSC, Human induced pluripotent stem cells; HCAECs, Human coronary artery endothelial cells; HPECs, Human placental endothelial cells; HUVECs, Human umbilical vein endothelial cells; IL, Interleukin; LPS, Lipopolysaccharide; MCP-1, Monocyte chemoattractant protein-1; MMP, Matrix metalloproteinase; PAI-1, Plasminogen activator inhibitor-1; PlGF-1, Placental growth factor-1; ROS, Reactive oxygen species; SGLT2i, Sodium–glucose cotransporter-2 inhibitors; TNF-α, Tumor necrosis factor-α; VE-cadherin, Vascular endothelial cadherin; VEGF-A, Vascular endothelial growth factor A; VSMCs, Vascular smooth muscle cells.

**Table 2:** Summary characteristics of the included human observational studies.

First Author, Date, Reference	Study Design	SGLT2i Therapy	Main Results
Seecheran, 2021 [39]	Diabetic patients with stable CAD; platelet function assessed with VerifyNow™ P2Y12 assay	Dapagliflozin	Significantly reduced P2Y12 reactivity (reaction units decreased by 20%)
Sardu, 2023 [44]	Diabetic patients with non-obstructive multivessel stable CAD	Commercially available SGLT2i, orally administered	Reduced macrophage grade and lipid arc; increased fibrous cap thickness (FCT) on OCT; lower MACE rate at 1-year follow-up
Kurozumi, 2024 [45]	Diabetic patients with ACS	Commercially available SGLT2i, orally administered	Increased FCT; reduced lipid arc; lower MACE and revascularization rates at follow-up
Zhang, 2024 [46]	Diabetic patients undergoing ≥2 coronary CT angiographies	Dapagliflozin (5 mg/day), Empagliflozin (10 mg/day), or Canagliflozin (100 mg/day)	Significantly reduced total plaque volume, primarily due to reduction in non-calcified plaque volume

Abbreviations: ACS, Acute coronary syndrome; CAD, Coronary artery disease; CT, Computed tomography; FCT, Fibrous cap thickness; MACE, Major adverse cardiovascular events; OCT, Optical coherence tomography; SGLT2i, Sodium–glucose cotransporter-2 inhibitors.

## Data Availability

No new data were created or analyzed in this study.

## Supplementary Materials

The following supporting information can be downloaded at: https://www.mdpi.com/article/10.3390/life15111784/s1, Methods S1: Full Search Strategy; Table S1: Comparative chemical and pharmacological properties of major SGLT2 inhibitors; Table S2: Quality assessment of in vitro and animal studies included in this systematic review; Table S3: Quality assessment of observational human studies included in this systematic review.

## Abbreviations

The following abbreviations are used in this manuscript:

ACS	Acute coronary syndrome
ApoE	Apolipoprotein E
CAD	Coronary artery disease
CRP	C-reactive protein
DM	Diabetes mellitus
EC	Endothelial cell
FC	Fibrous cap
IL	Interleukin
MACE	Major adverse cardiovascular events
MCP-1	Monocyte chemotactic protein 1
NHE1	Sodium-hydrogen exchanger
NLRP3	Nucleotide-binding Oligomerization Domain-Like Receptor Protein 3
NO	Nitric oxide
OCT	Optical coherence tomography
ROS	Reactive oxygen species
SGLT2i	Sodium-glucose cotransporter-2 inhibitors
TNF-α	tumor necrosis factor-α
VSMCs	Vascular smooth muscle cells
