# Supplementary material for: The Impact of Sodium Glucose Co-Transporter 2 (SGLT-2) Inhibitors on Atherogenesis: A Systematic Review of Experimental and Clinical Evidence
Source: Life (Basel). 2025 Nov 20;15(11):1784. doi: 10.3390/life15111784 (PMC12653638; doi:10.3390/life15111784)
Supplement: Supplementary file 1 [file life-15-01784-s001.zip › life-3976532-supplementary.pdf]

Supplementary Methods S1. Full search strategy.

MEDLINE/PubMed: ((Sodium-glucose co-transporter[Title] OR Sodium glucose cotransporter[Title] OR SGLT2[Title] OR dapaglifozin[Title] OR empaglifozin[Title] OR canaglifozin[Title]) AND (atheroscler*[Title/abstract] OR plaque[Title/abstract] OR coronary[Title/abstract]) AND (phenotype[Title/abstract] OR fibrous cap thickness[Title/abstract] OR macrophage[Title/abstract] OR lipid[Title/abstract] OR calcium[Title/abstract] OR vulnerability[Title/abstract] OR stability[Title/abstract] OR instability[Title/abstract] OR endothelial[Title/abstract]))
EMBASE: (‘sodium-glucose co-transporter’:ti OR ‘sodium glucose cotransporter’:ti OR SGLT2:ti OR dapaglifozin:ti OR empaglifozin:ti OR canaglifozin:ti) AND (atheroscler*:ab,ti OR plaque:ab,ti OR coronary:ti) AND (phenotype:ab,ti OR fibrous cap thickness:ab,ti OR macrophage:ab,ti OR lipid:ab,ti OR calcium:ab,ti OR vulnerability:ab,ti OR stability:ab,ti OR instability:ab,ti OR endothelial:ab,ti)

SCOPUS: TITLE/ABSTRACT (“sodium-glucose co-transporter” OR “sodium glucose cotransporter” OR SGLT2 OR dapaglifozin OR empaglifozin OR canaglifozin) AND (atheroscler* OR plaque OR coronary) AND (phenotype OR fibrous cap thickness OR macrophage OR lipid OR calcium OR vulnerability OR stability OR instability OR endothelial)

WEB OF SCIENCE: TI=(“sodium-glucose co-transporter” OR “sodium glucose cotransporter” OR SGLT2 OR dapaglifozin OR empaglifozin OR canaglifozin) AND TI=(atheroscler* OR plaque OR coronary) AND TS=(phenotype OR fibrous cap thickness OR macrophage OR lipid OR calcium OR vulnerability OR stability OR instability OR endothelial)

GOOGLE SCHOLAR: allintitle: SGLT2 AND atherosclerosis

Supplementary Table S1. Comparative chemical and pharmacological properties of major SGLT2 inhibitors

	**Dapagliflozin**	**Empagliflozin**	**Canagliflozin**
Chemical class	C-aryl β-D-glucopyranoside	C-aryl β-D-glucopyranoside	C-aryl β-D-glucopyranoside
Unique substituent (aglycone)	Ethoxy-benzyl group	Tetrahydrofuran-oxy benzyl group	Thiophene-benzyl group
SGLT2 / SGLT1 selectivity ratio	≈1200	≈2500	≈250
Oral bioavailability (%)	78	60	65
Time to peak plasma (Tmax)	1–2 h	1.5 h	1–2 h
Elimination half-life (t½)	≈12.9 h	≈12.4 h	≈10.6 h
Metabolism	Glucuronidation (UGT1A9 > UGT2B4)	Glucuronidation (UGT2B7)	Glucuronidation (UGT1A9, UGT2B4)
Primary excretion route	Renal excretion	Renal excretion	Renal excretion

Supplementary Table S2. Quality assessment of in vitro and animal studies included in this systematic review

**First Author, Date, Ref**	**1**	**2**	**3**	**4**	**5**	**6**	**7**	**8**	**9**	**10**	**11**
Li, 2021 [21]											
Pawlos, 2023 [22]											
Semo, 2023 [20]											
Lin, 2025 [24]											
Ganbaatar, 2020 [25]											
Guo, 2023 [23]											
Terasaki, 2015 [26]											
Terasaki, 2017 [27]											
Pennig, 2019 [28]											
Chen, 2023 [29]											
Chen, 2022 [30]											
Leng, 2016 [31]											
Han, 2017 [33]											
Nakatsu, 2017 [32]											
Lee, 2020 [34]											
Iwamoto, 2022 [35]											
Xu, 2024 [36]											
Lin, 2014 [37]											
Spigoni, 2020 [38]											
Liberale, 2023 [40]											
Chen, 2023 [41]											
Wu, 2024 [42]											

Li, 2024 [43]	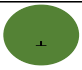	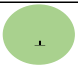	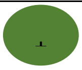	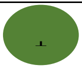	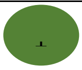	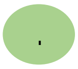	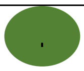	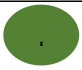	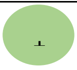	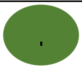	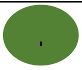

Table legend: Ref.: reference; 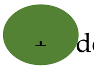 definitively low; 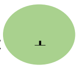 : probably low; : probably high; 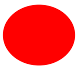 : definitely high

Domains: 1. Randomization of animals or experimental units; 2. Allocation concealment; 3. Adequacy of experimental control groups; 4. Blinding of researchers during exposure and outcome assessment; 5. Similarity of experimental conditions across groups; 6. Completeness of outcome data; 7. Selective outcome reporting; 8. Exposure characterization; 9. Outcome assessment methods; 10. Statistical analysis and power; 11. Other potential sources of bias.

Supplementary Table S3. Quality assessment of observational human studies included in this systematic review

First Author, Date, Ref	1	2	3	4	5	6	7	8	9	10	11	12	13	14
Seecheran, 2021 [39]	Y	Y	Y	Y	N	Y	Y	/	Y	/	Y	N	Y	Y
Sardu, 2023 [44]	Y	Y	Y	Y	N	Y	Y	/	Y	/	Y	N	Y	Y
Kurozumi, 2024 [45]	Y	Y	Y	Y	N	Y	Y	/	Y	/	Y	N	Y	Y
Zhang [46]	Y	Y	Y	Y	N	Y	Y	/	Y	/	Y	N	Y	Y

Table legend: Ref.: reference. Y = yes; N = no; /: not applicable; ?: cannot be determined.

Domains: 1. Research question clearly stated; 2. Study population clearly specified and defined; 3. Participation rate of eligible persons at least 50%; 4. Subjects recruited from the same or similar populations; 5. Sample size justification or power description; 6. Exposure assessed prior to outcome measurement; 7. Sufficient timeframe to observe an association; 8. Different levels of exposure assessed; 9. Exposure measures clearly defined, valid, and reliable; 10. Outcome measures clearly defined, valid, and reliable; 11. Blinding of outcome assessors; 12. Loss to follow-up after baseline 20% or less; 13. Confounding variables measured and adjusted statistically; 14. Other potential threats to internal validity considered.
